# Detecting microRNAs of high influence on protein functional interaction networks: a prostate cancer case study

**DOI:** 10.1186/1752-0509-6-112

**Published:** 2012-08-28

**Authors:** Mohammed Alshalalfa, Gary D Bader, Anna Goldenberg, Quaid Morris, Reda Alhajj

**Affiliations:** 1 Department of Computer Science, University of Calgary, Calgary, AB, Canada; 2 University of Toronto, and the Department of Molecular Genetics, University of Toronto, Toronto ON, Canada; 3 Genetics and Genome Biology, Toronto, Canada; 4 Biotechnology Research Centre, Palestine Polytechnic University, Hebron, Palestine

**Keywords:** MiRNA, Protein interactions, Systems biology, High-influence miRNA

## Abstract

**Background:**

The use of biological molecular network information for diagnostic and prognostic purposes and elucidation of molecular disease mechanism is a key objective in systems biomedicine. The network of regulatory miRNA-target and functional protein interactions is a rich source of information to elucidate the function and the prognostic value of miRNAs in cancer. The objective of this study is to identify miRNAs that have high influence on target protein complexes in prostate cancer as a case study. This could provide biomarkers or therapeutic targets relevant for prostate cancer treatment.

**Results:**

Our findings demonstrate that a miRNA’s functional role can be explained by its target protein connectivity within a physical and functional interaction network. To detect miRNAs with high influence on target protein modules, we integrated miRNA and mRNA expression profiles with a sequence based miRNA-target network and human functional and physical protein interactions (FPI). miRNAs with high influence on target protein complexes play a role in prostate cancer progression and are promising diagnostic or prognostic biomarkers. We uncovered several miRNA-regulated protein modules which were enriched in focal adhesion and prostate cancer genes. Several miRNAs such as miR-96, miR-182, and miR-143 demonstrated high influence on their target protein complexes and could explain most of the gene expression changes in our analyzed prostate cancer data set.

**Conclusions:**

We describe a novel method to identify active miRNA-target modules relevant to prostate cancer progression and outcome. miRNAs with high influence on protein networks are valuable biomarkers that can be used in clinical investigations for prostate cancer treatment.

## Background

A major challenge in biomedical research is to understand the underlying mechanisms of human disease. Great effort has been spent on determining genes associated with human diseases. However, most human diseases, and cancer in particular, cannot be attributed to single gene but arise due to complex interactions among multiple components of the cell, including genes, proteins, and miRNAs [1]. miRNAs are a large family of gene regulators, found in both plants and animals, which impact gene activity by binding to the 3’UTR of target mRNAs leading to mRNA degradation or translational inhibition [2,3]. Though miRNAs are only 18-22 nucleotides, each can control the expression of hundreds of genes. It is estimated that approximately half of the human genome is regulated by miRNA-mediated gene control [4]. miRNAs play a key role in regulating diverse cellular functions, such as development, proliferation, apoptosis, and metabolism [2] and are associated with a growing list of diseases including cancer [5,6]. An increasing body of evidence suggests that miRNAs impact gene expression in many cancer types including prostate cancer [7-9]. Several studies have investigated the role of miRNAs in cancer using mRNA and miRNA expression profiling [3,10]. Better understanding the regulatory role of miRNAs in cancer development and progression requires exploring their influence on other components of the cellular system they are a part of. Doing so, may lead to identifying predictive biomarkers and developing novel therapeutic strategies for cancer.

Current major challenges in miRNA research are prediction and experimental validation of miRNA-target interactions, and determination of the functional role of miRNAs. Computational prediction of miRNAs is challenging in human genomes because of the imperfect pairing of the miRNA with the corresponding target site [11]. Several factors can influence miRNA-mediated gene control, like 3’UTR length, number of miRNA targets sites [11], degree of complementary match [12], amount of target mRNA [12,13], and the competition for targeted mRNA [14]. Unfortunately, current sequence based predictions produce many false positive interactions and many of the predicted interactions may not be functional [15], which means there may be no relationship between the expression levels of the mRNA and the predicted targeting miRNA. Several studies have tried to solve this by integrating gene expression data with sequence-based prediction to remove non-functional interactions and keep interactions that show negative correlation between miRNA and their targets [10,16]. Thus, sequence-based methods provide a general view of the potential miRNA targets but expression data or other cellular context information is required to more accurately predict miRNA-target interactions.

Determining the role of individual miRNAs in cellular regulatory processes is still a major challenge. The function of many miRNAs remains unknown, and even for relatively well studied miRNAs, only a handful of their targets have been characterized [17,18]. Delineating miRNA function through knock-out and overexpression experiments in model organisms has had limited success, possibly because of functional redundancy among miRNAs or among gene pathways regulated by miRNAs [19]. A miRNA downregulates its targets, thus negative correlation in expression levels between a miRNA and its direct targets indicates that the miRNA is functional. Several studies have attempted to extract miRNA-target modules based on the correlation between miRNAs and targets [20] and based on graph theory [21]. However, these results are complicated by indirect effects - a single miRNA may target many mRNA targets that may influence other genes, thus negative correlation between miRNA and targets does not indicate a direct interaction between miRNA and target.

Interactions between miRNA and targets are not solely dependent on the 3’UTR of the target, but depend on what other competing 3’UTR targets are expressed in a given cellular context. Limited attempts have been made to investigate the impact of miRNAs on protein interactors of the target. It has been shown that protein-protein interaction (PPI) network topological features help to filter out false positive targets [22], and help to prioritize miRNAs in prostate cancer [23]. Recent evidence showed that some protein complexes are enriched with single miRNA targets and some complexes are enriched with miRNA cluster targets [24]. For example, SMAD3-SMAD4-FOXO3 complex is enriched with miR-1284 targets, and MAD1-SIN3A-HDAC2 complex is enriched with targets of the miR-510-514 and miR-1912-1264 clusters. Other studies demonstrated that PPI context of miRNA targets provides more representative information about miRNA function compared to using only direct targets [25]. Direct targets of miRNAs and their partners jointly showed higher modularity levels compared with miRNA direct targets alone [25]. Analyzing properties of miRNA targets is a promising approach to miRNA function prediction. mirPath [26] is a computational tool developed to identify molecular pathways enriched in miRNA targets set. mirPath extracts miRNA targets from other tools such as TargetScan [27], PITA [28], and then miRNA function is predicted by assessing whether the predicted targets of a given miRNA are enriched for particular functional annotations. Such enrichment based methods suffer from several limitations. First, they solely depend on the miRNA-target prediction algorithms that are noisy. Second, predicted miRNA targets are usually large (hundreds to thousands of genes) and this leads to heterogeneous functional annotations that make it difficult to gain high confidence predictions. Integrating expression data is a promising approach to reduce noise in enrichment results. The miRNA body map [29] is a web tool developed for miRNA functional annotation in normal and diseased human tissues that integrates expression data to reduce heterogeneity in functional annotations. FAME [30] is another tool with three main applications in the area of miRNA functional analysis. Firstly, it infers miRNA function directly using sets of genes sharing common annotations and secondly, infers miRNA function indirectly using matched mRNA/miRNA expression data. Thirdly, FAME predicts the function of genomic clusters of miRNAs. Integrating the protein context of miRNA targets is another promising dimension for miRNA function prediction. miRUPnet [31] is another miRNA function prediction framework that predicts miRNA function based on the upstream context of miRNA and not downstream. It infers the miRNA function by functionally analyzing the context of its transcription factors in a protein-protein interaction network. Using information about TFs upstream of a miRNA results in the discovery of additional biological processes not seen in miRNA targets (downstream). These observations shed light on the influence of miRNAs on the PPI subnetwork involving the targets, and highlight the importance of considering target protein interactors when searching for functional miRNA-target interactions.

In the post-genomics era, a crucial task in molecular biology is to understand miRNA regulation in the context of biological networks. Since miRNAs target proteins that are part of either protein complexes or signaling pathways, it is important to study the influence of miRNAs on protein networks in disease progression. Characterizing the role of miRNAs in the context of protein networks has emerged recently in several studies [25,32-34]. By analyzing the interactions between miRNAs and cellular signaling networks, miRNAs were found to predominantly target proteins of the same signaling pathway and target highly connected scaffolds and most downstream network components such as signaling transcription factors. miRNAs were also found to less frequently target upstream components of the signaling pathways like membrane receptors and ligands [34]. Hsu *et al*[25] demonstrated that many miRNA-targeted genes are hub proteins and bottleneck proteins in protein interaction networks (PPIN) and thus have higher betweeness centrality. When these hub or bottleneck proteins are repressed by individual or multiple miRNAs, they may consequently influence large part of the interacting proteins and thus control key components of the PPIN. Their analysis showed that the target proteins of individual miRNAs tend to interact with more proteins than other non-miRNA targets. Positive correlation between protein connectivity (degree in PPIN) and the number of miRNAs targeting the corresponding protein has been observed by Liang and Li [32]. This means that proteins with large numbers of partners in the PPIN network need more miRNAs to control their expression. miRNA induced influence can propagate in the regulatory network by targeting master transcription factors. Cui *et al*[33] found that 42% of 9348 gene that are regulated by TFs, are miRNA targets, and the average TF binding site count of miRNA targets is significantly higher than that of non miRNA targets. This suggests that gene expression regulation by miRNAs at the post-transcriptional level is coordinated with that of TFs at the transcriptional level and genes targeted by more miRNAs have more TF binding sites.

In this work we introduce a new method to characterize miRNA function based on its effect on the expression of the target and its neighbors in a functional interaction network. Unlike previous methods that weight miRNA-target interactions based on sequence complementarity or gene expression correlation alone, we estimate the overall influence of a miRNA on its target based on the target gene expression level and the gene expression levels of the interaction neighborhood of the target. miRNAs with high influence are validated using independent miRNA expression datasets, and by analyzing the biological pathway enrichment of target protein modules. We then used our miRNA-target influence network to predict the overall influence of each miRNA on individual prostate cancer patients to find those miRNAs associated with aggressive cancer. We show that miRNAs with high influence on protein complexes and biological processes are likely involved in cancer progression and have potential prognostic significance.

## Methods

### miRNA targets

Human miRNA target predictions for miRNA with conserved 3’UTR were taken from TargetScan 5.1 [27], and experimentally validated miRNA and their targets were taken from mirTarBase [35] and miRecord [36]. We used the union of mirTarBase and miRecord as a source of experimentally validated miRNA-target interactions(3976 interactions between 345 miRNA and 2277 gene).

### Functional protein interaction (FPI) networks

We used combined undirected functional protein interactions (FPI) as described in [37]. FPI includes annotated functional protein interactions from Reactome, Panther, CellMap, BioCarta, KEGG and TRED, and includes interactions derived from physical protein interaction, co-expression data, domain-domain interaction data. FPI was constructed using a naive Bayes classifier (NBC) to distinguish high-likelihood FIs from non-functional pairwise relationships. We also used physical protein interactions from the HPRD database [38]. FPI functional interaction network includes HPRD interactions, but the two networks have distinct topological features. We also used another curated human signaling network from Cui *et al*[39].

### miRNA and mRNA expression data

We used mRNA and miRNA expression data from the MSKCC Prostate Oncogenome Project (Taylor data)that is available at the Gene Expression Omnibus (GEO accession number: GSE21032) [40]. The data contains expression levels of 26443 genes across 179 samples (131 primary cancer, 19 metastatic, and 29 normal samples), and expression of 370 miRNAs across 140 samples. We used the expression data of 139 samples with both mRNA and miRNA data for our analysis. To validate the miRNA results we obtained using the Taylor data, we used localized prostate cancer miRNA expression data from independent prostate patient cohort (GSE23022 [41]) and prostate cell lines (NCI60) [42].

### miRNA-target influence(miRTI) network construction

The initial miRNA-target network is defined by a sequence based search (*Seq*); *Seq*(*miR*,*t*) = 1, if *t* is a potential target for *miR*based on the TargetScan conserved sequence based prediction, otherwise *Seq*(*miR*,*t*) = 0. The relationship between the miRNA expression level (miR) and that of its (t) was computed using mutual information:

(1) $$MI_{\text{miR},t}=\underset{d\in \text{miR}}{\sum }\underset{r\in t}{\sum }p(d,r)\log\left(\frac{p(d,r)}{p(d)p(r)}\right)$$

*p*(*d*,*r*) is the joint probability density function(pdf) of *miR* and *t*, and P(d) and p(r) are the marginal pdf’s of *miR*and *t* respectively.

We propose that the influence of a miRNA (*miR*) on its target(*t*) depends on three variables. First is the strength of the negative correlation between miRNA and target expression profiles. *CorrmiR*(*miR*,*t*) =* MI*(*miR*,*t*), if *Seq*(*miR*,*t*) = 1, and *CorrmiR*(*miR*,*t*) = 0, if *Seq*(*miR*,*t*) = 0. We only considered *miR* and *t* pairs with negative Pearson’s correlation and *Seq*(*miR*,*t*) = 1. This step is needed to filter out miRNA-target pairs with high *MI* due to positive correlation.

Second is the direct impact of the miRNA on the expression of the partners of the target. We calculated mutual information (*MI*) between the expression profiles of each target and its FPI partners (*CorrFPI*), where *CorrFPI*(*t*_*i*_,*t*_*j*_) =* MI *(*t*_*i*_,*t*_*j*_) if *t*_*i*_ is linked to *t*_*j*_ in FPI, and *CorrFPI*(*t*_*i*_,*t*_*j*_) = 0 otherwise. We used the maximum MI between the target and its partners to represent the direct influence of miRNA on the target partners.

Third is the indirect influence of miRNA on the expression of the target through its partners. The indirect impact of a miRNA on its target through its partners is defined as *W *, where $W(\text{miR},t)=\underset{k}{\sum }\text{MI}(\text{miR},k)\times \text{MI}(k,t)$ where *k* is the partners of *t* in FPI.

**Figure 1:**
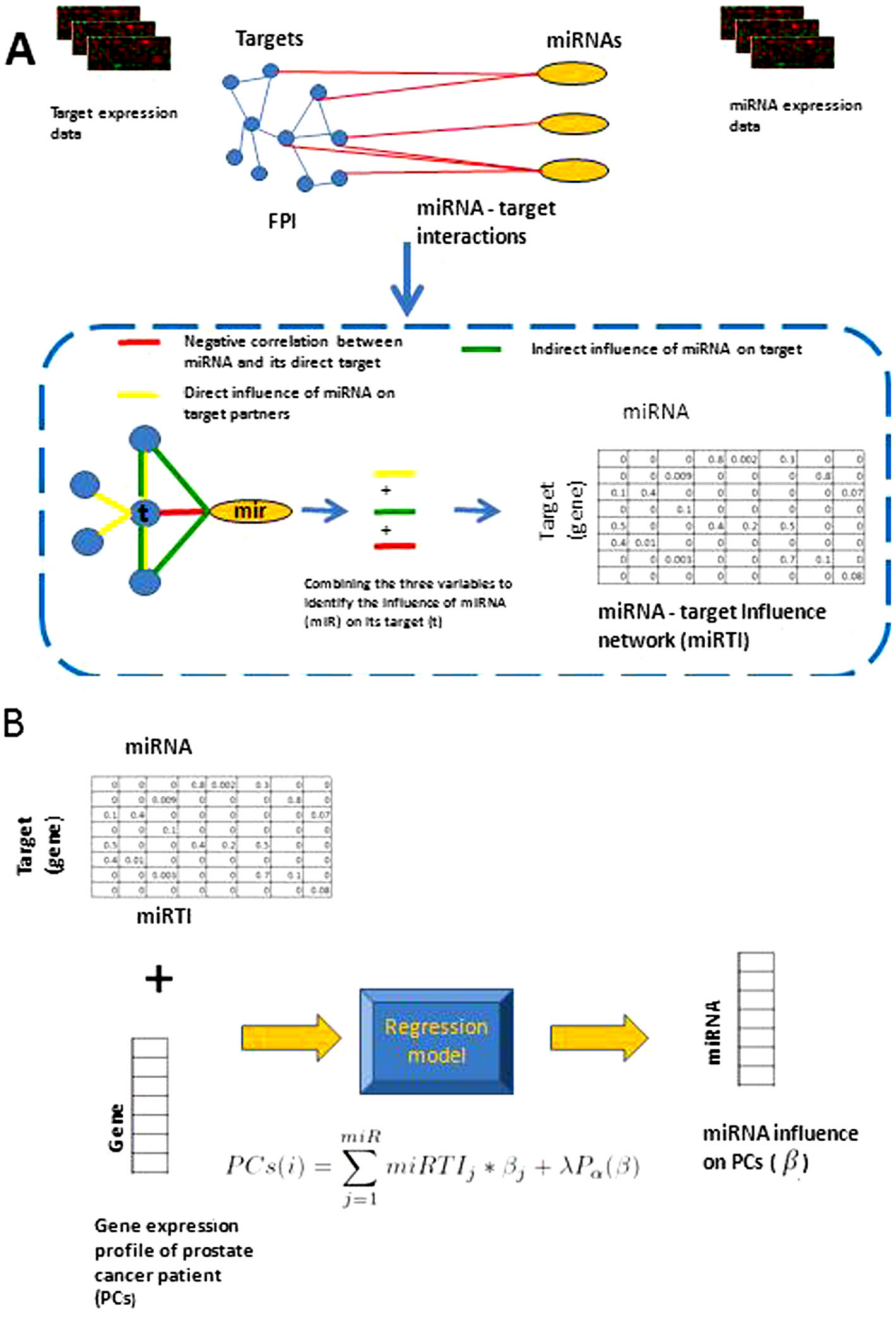
**Overview of constructing miRNA-target influence (miRTI) network and its application. A**. miRTI is constructed by integrating matched miRNA and mRNA expression data with FPI and miRNA-target networks. The influence of a miRNA on its target is based on combining three variables; strength of negative correlation between miRNa and its target, direct influence of miRNA on target partners and indirect influence of miRNA on target through partners. **B**. After constructing the miRTI, we used it to predict miRNA-patient influence network using elastic-net regression model. The gene expression profile of each PCs is a linear combination of the influence of miRNAs on its targets.

We assess the influence of a miRNA (*miR*) on its potential target mRNA (*t*) by integrating these three evidences. Figure 1A provides a schematic description of integrating gene expression and FPI to identify miRNA influence on a protein target. The *miRTI* network was calculated by combining the three evidences of association between miRNA(*miR*) and its targets(*t*) as:

(2) $$\begin{array}{l} \text{miRTI}(\text{miR},t)=\text{CorrmiR}\text{miR}(,t)*W(\text{miR},t)\;\\ \;\;\;\;*\text{MAX}(\text{CorrFPI}(t,k)) \end{array}$$

### Using the miRNA-target influence(miRTI) network to measure the influence of miRNAs on prostate cancer progression

We used the *miRTI* network to predict miRNAs with influence on gene expression profiles of prostate cancer samples (PCs). We model the gene expression of prostate cancer samples as a linear combination of miRNA effects on their targets [43], as follows:

(3) $$\text{PCs}(i)=\overset{\text{miR}}{\underset{j=1}{\sum }}\text{miRT}I_{j}*\beta_{j}+\lambda P_{\alpha }(\beta )$$

where

(4) $$P_{\alpha }(\beta )=\overset{\text{miR}}{\underset{j=1}{\sum }}[\frac{1}{2}(1-\alpha )\beta_{j}^{2}+\alpha |\beta_{j}|]$$

is the elastic-net penalty. *P*_*α*_ is a compromise between the ridge regression penalty (*α *= 0), and the lasso penalty (*α *= 1). In this model we set *α* to be 0.5 as a middle value between (*α *= 0)(ridge regression) and (*α *= 1) (lasso regression). Setting (*α *= 0.5) will reduce sparsity achieved using lasso regression while still panelizing correlated predictors. This penalty is particularly useful when there are many correlated predictor variables as in the case of miRNAs. We tried several values of *α* and we saw that when *α *changes from 0 to 0.5 or 1, it dramatically changes the minimum *λ* value and the number of non-zero values in the solution. Setting *α *to 0 produced large number of non-zero values in the solution (113) and *α *= 1 produced small number of non-zero values (11) that lead to a very sparse solution that might affect predictors of small effect on the response. Setting *α *to 0.5 leads to a solution of medium sparsity with 31 non-zero elements (Additional file 1: Figure S1). However, changing *α* around 0.5 (0.3-0.7) does not change minimum *λ *value a lot (difference is 0.01) which does not impact number of non-zero elements. We have selected *α* to be 0.5 because when it is 0.5, the minimum *λ* leads to the minimum MSE. *β *is the regression coefficient of each variable, which indicates how the expression level of each miRNA can explain the gene expression profile of prostate cancer samples. *λ* is a factor that determines the sparsity of the solution, as *λ* increases, the number of nonzero components of *β* decreases. In this study, we selected 100 values of *λ *and used those that minimize the mean square error. More details on *λ* optimization with respect to *α *is shown in Additional file 1: Figure S1. Elastic-net regression was fit using ten-fold cross validation. We used glmnet package available at http://www-stat.stanford.edu/ tibs/glmnet-matlab/ to solve the regression model. For each patient we predicted the influence of the miRNA set on the patient’s gene expression profile. Figure 1B describes the steps to construct the input of the model and its output. The resulting miRNA-patient influence profile was used to associate a miRNA with a sample’s outcome.

### Detection of transcriptional activity centers in prostate cancer

Several studies have shown that a functional interaction network provides information about the function of a gene of interest using the guilt by association concept [44,45]. We define activity score for each gene based on the importance of the neighbor genes, similar to other studies [46]. First, we computed the differential expression significance of each gene (*R*) in the prostate cancer gene expression data using the Student’s t-test. The bigger *R* is, the more significant the expression of the gene is. We then used the prostate cancer *CorrFPI* network to define the strength of the relationship between the gene of interest and its neighbors in the FPI network. *ActivityScore* for *gene*(*i*) is defined as:

(5) $$\begin{array}{l} \;\text{ActivityScore}(i)=[\frac{1}{N}\overset{N}{\underset{d=1}{\sum }}\text{CorrFPI}(i,d)\;\\ \;\;\;\;\;*R(d)]*\;\text{MAX}(\text{CorrFPI}(i,d)*R(d))\; \end{array}$$

Where *N* is the total number of neighbor genes.

### Using the miRNA-target influence(miRTI) network to identify miRNA influence on genes with high activity center scores

The *ActivityScore* measure reflects transcriptional activity of genes in modules rather than single genes. Here we model the expression activity of genes as a linear combination of the miRNAs’ expression. We used the miRTI network that represents the influence of each miRNA on each genes activity centers score to predict miRNAs that explain the transcriptional activity center score using the same regression model as used above:

(6) $$\text{ActivityScore}(i)=\overset{\text{miR}}{\underset{j=1}{\sum }}\text{miRT}I_{j}*\beta_{j}+\lambda P_{\alpha }(\beta )$$

The output of this model is a coefficient for each miRNA that represents how each miRNA explains the expression activity score of the genes.

## Results

### Global correlation between functional protein network topology and miRNA regulation

**Figure 2:**
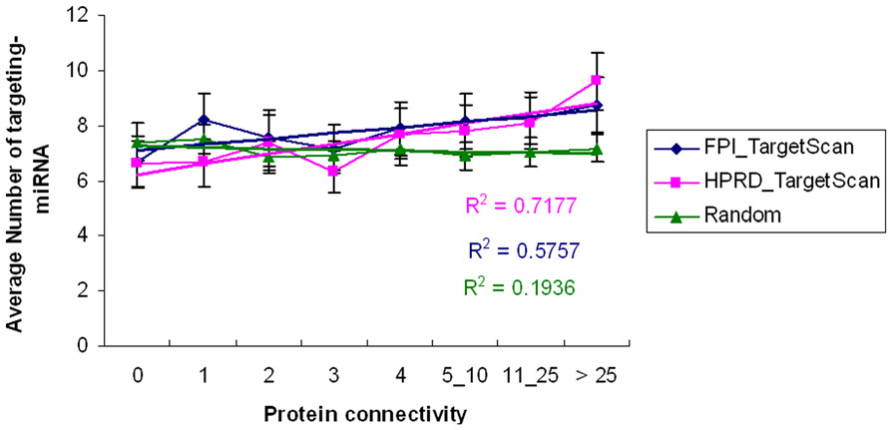
**Positive correlation between protein connectivity and number of miRNAs targeting the protein.** We used FPI and HPRD protein networks and TargetScan predictions to assess the correlation. Protein networks and TargetScan predictions are noisy and incomplete, therefore we randomized the protein network and miRNA-target networks to ensure that the positive correlation is not due noise.

To gain a global view of miRNA regulation of the FPI network, we analyzed the relationship between the FPI network topological features and miRNA regulation using two protein-protein interaction datasets (FPI, HPRD) and two miRNA-target networks (TargetScan) and miRecord (union of miRecord and mirTarBase known targets). We found a strong positive correlation between protein connectivity and the number of miRNAs targeting the corresponding protein (Figure 2) and a negative correlation between protein clustering coefficient and number of targeting miRNAs (Table 1), across all networks we analyzed. Other network topological measures, like betweenness, did not show significant correlation. This means that proteins with large numbers of partners in the FPI network need more miRNAs to control their expression, and protein modules, such as complexes that are highly connected (thus have high clustering coefficients) need a smaller number of miRNAs. We also performed this analysis on randomly generated protein and miRNA-target networks and did not observe any significant trend. These results are in agreement with a recent studies by Liang and Li [32] who found positive correlation between protein connectivity and average number of miRNA target site types. These findings motivated us to consider the protein network when estimating the influence of a miRNA on its target.

**Table 1:** Correlation between protein network structure and miRNA activity

	**Protein connectivity**	**Clustering coefficient**
	*R*^2^ (p-value)	*R*^2^ (p-value)
FPI-TargetScan	0.73 (0.004)	-0.77 (0.01)
HPRD-TargetScan	0.85 (0.001)	-0.28 (0.2)
FPI-miRecord	0.80 (0.002)	-0.148(0.8)
HPRD-miRecord	0.64 (0.009)	-0.537(0.02)
Random	-0.26 (0.54)	0.22 (0.5)

Spearman’s rank correlation of protein connectivity and clustering coefficient vs. number of targeting miRNAs, significance of correlation is indicated by p-value in brackets, FDR < 0.005.

### Prostate cancer miRNAs target functionally associated genes

**Figure 3:**
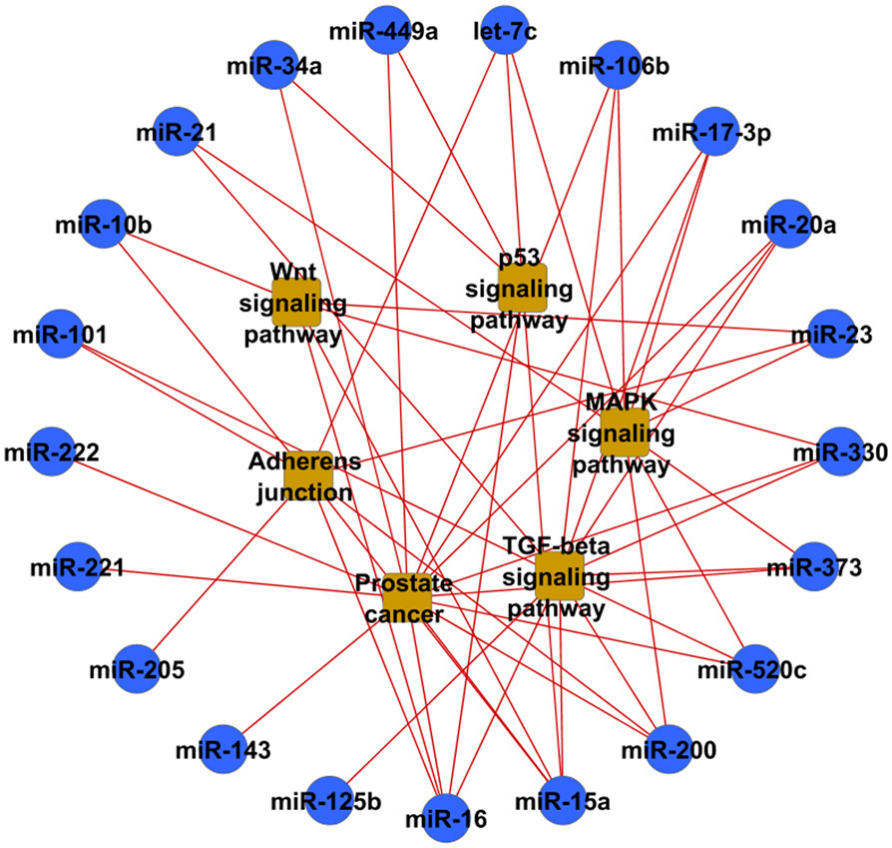
**Prostate cancer miRNA targets are enriched with pathways associated with cancer.** We used TargetScan for miRNA target prediction and DAVID for target pathway enrichment analysis. Links between miRNAs and pathways indicate that the Benjamini corrected enrichment p-value is less than 5 × 10^−5^.

**Figure 4:**
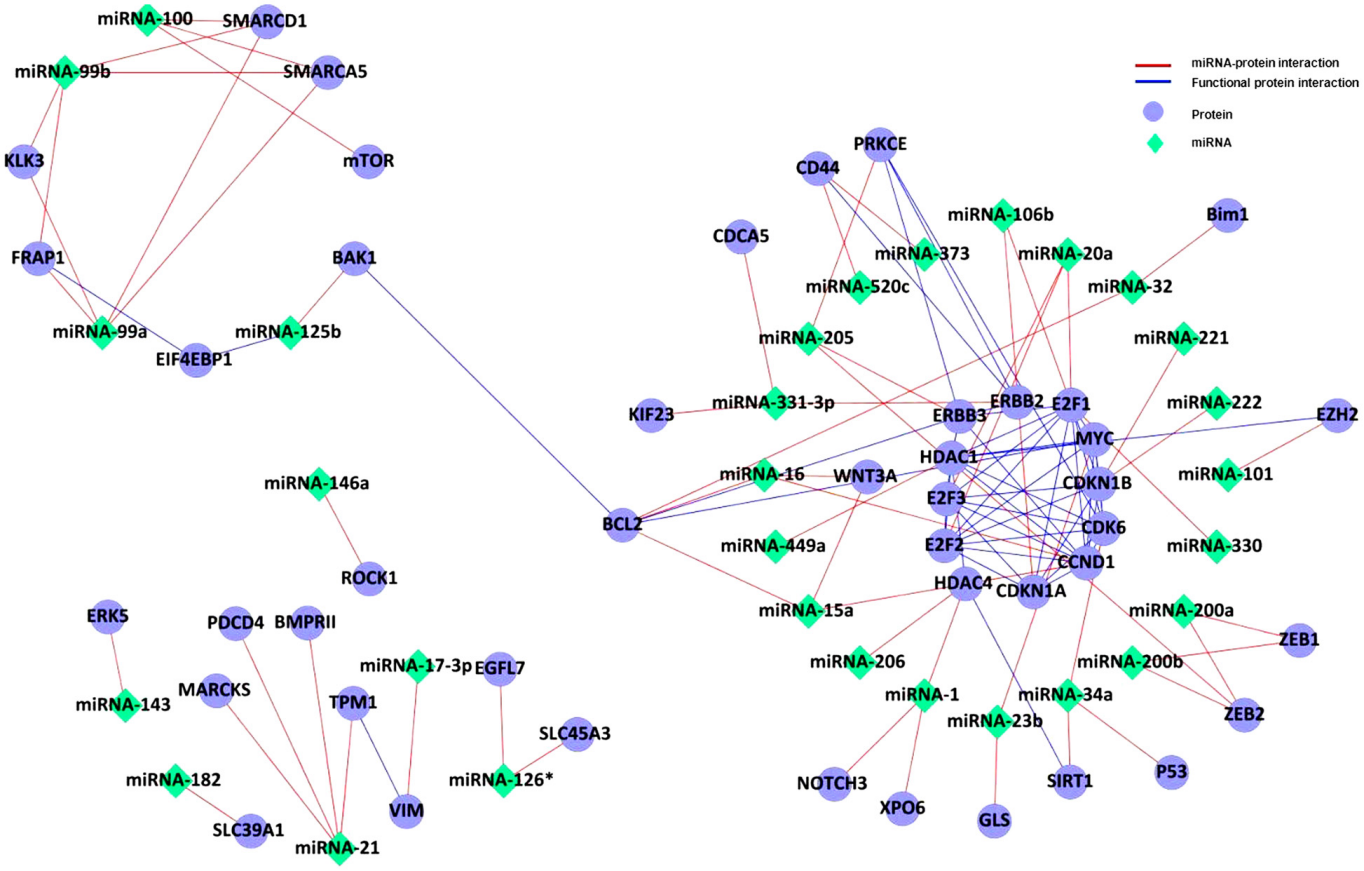
**Prostate cancer miRNAs target functionally related proteins.** Targets of miRNAs that play a role in prostate cancer progression showed to be functionally related. Most of the miRNAs target proteins related to histone deacytelation and cadherin pathways.

We manually collected a list of 54 miRNAs that were experimentally validated to play a role in prostate cancer from several piblushed sources (Additional file 1). 30 out of 54 have known targets as shown in Table S1. We also extracted 132 experimentally supported prostate miRNAs from miR2Disease [47] and HMDD [48] databases. We next asked if the targets of the experimentally validated prostate miRNAs are functionally related. For each miRNA, we predicted its targets using TargetScan and then performed pathway enrichment analysis on the targets using the DAVID online software ( http://david.abcc.ncifcrf.gov/). Enrichment analysis results (Figure 3) revealed that prostate related miRNAs target proteins that are often in the same pathways and are functionally related. Figure 4 shows that the corresponding miRNA targets are functionally related and connected in protein networks. This motivated us to consider the target protein context to assess the influence of miRNAs.

### The FPI network helps reveal miRNA-target modules that play a functional role in prostate cancer

**Figure 5:**
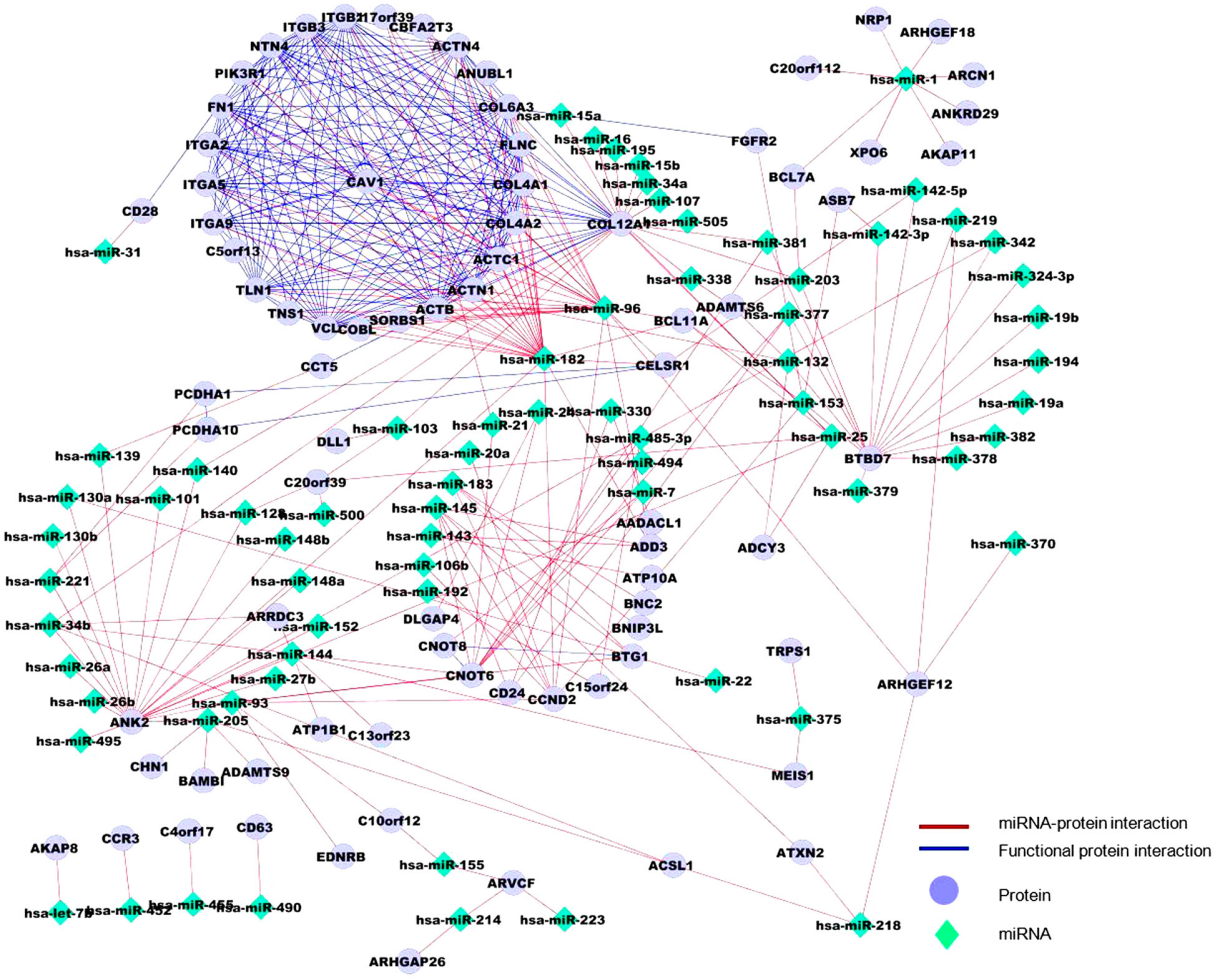
**Functional miRNA-target network extracted based on the influence of miRNAs on target protein complexes.** A miRNA-target link (red) indicates that the miRNA (diamond) influences the gene expression of the target and proteins functionally interacting with it (circles). Links between two targets (blue) indicate that they are interacting in the FPI network. Cytoscape [49] was used for network visualization.

**Figure 6:**
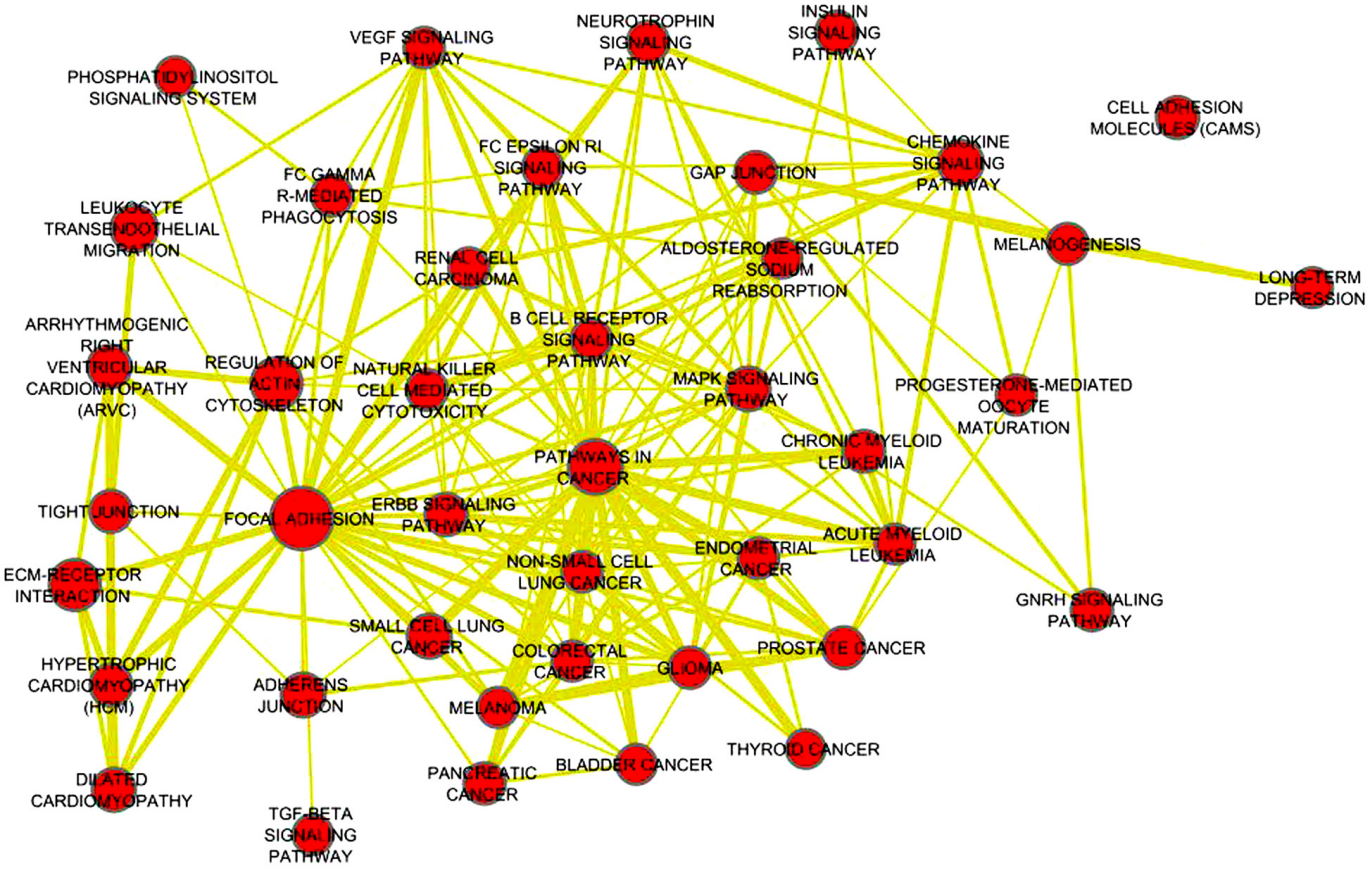
**Pathway enrichment map **[50]** of first degree partners of targets of functional miRNAs predicted in Figure**3**.** A. Nodes represent significant enriched pathways, links indicate the gene overlap between pathways. Node size represents the pathway enrichment significance, the larger the node, the more significant the enrichment pathway is. Results revealed that the targets of miRNAs and their partners are highly associated with focal adhesion and path-ways in cancer.

We identified miRNAs that are functionally active in prostate cancer, i.e. they have a high influence on the protein partners of their targets using the miRNA-target influence (*miRTI*) network that was constructed based on prostate cancer expression data and FPI network. We selected the top miRNA-target interactions (Figure 5) with values greater than the upper quartile. We observed that the targets of high influence miRNAs are functionally related, which could explain, at a high-level, the mode of action of miRNAs. To investigate the functionality of the predicted miRNA-target modules and further assess the robustness of our predictions, we followed two tracks. First, the enriched pathways of miRNA targets (81 proteins) were characterized. Enrichment analysis revealed that predicted targets are highly associated with focal adhesion and prostate cancer pathways (p-value < 1 × 10^−20^). As a control, we compared the predicted *miRTI* network (Figure 5) that was generated using Cytoscape [49] with the *Corrmir* network (Additional file 1: Figure S2), which only represents correlation between miRNAs and their target without considering the downstream regulation of miRNAs on FPI target neighbors. Targets in *Corrmir* also were enriched in focal adhesion and prostate cancer, but not as significantly (p-value < 1 × 10^−11^). We also randomly generated a functional miRNA-target network by randomizing the FPI and the Seq miRNA-target networks and target did not show any significant enrichment. In the second track, we extracted the expression profile of miRNAs predicted to have high influence in two independent prostate cancer miRNA expression datasets prostate cell line NCI60 [42], and a prostate patient cohort (GSE23022 [41]). Here, it is worth mentioning that we do not extract differentially expressed genes from NCI60 and GSE23022, but rather we only extract the expression of our miRNA list predicted to have high influence on targets. In both datasets, miRNAs predicted to have high influence on target interactors demonstrated diagnostic and prognostic significance. miRNAs predicted to have high influence based on our approach (70 miRNAs) are associated with prostate cancer better than those derived from the *Corrmir* network and miRNA-target network generated from the random FPI network based on multiple functional analysis strategies. First, a large proportion of predicted high influence miRNAs (29 out of 70 based on our 54 prostate miRNA list, and 40 out of 70 based on the miRNAs extracted from HMDD and miR2Disease) are known to play a role in prostate cancer development (Table S1). Several oncogene miRNAs like miR-221, miR21, miR-125b, and miR-106b were identified in addition to several tumor suppressor miRNAs like miR-34a, miR-20a, miR-1, miR15a and miR-16 [3,5,9]. Second, high influence miRNAs are better able to accurately discriminate prostate cancer from normal samples. miRNAs with high-influence on protein complexes were able to classify patients in the Taylor data [40] into normal vs. cancer patients with 97% classification accuracy using a linear SVM, better than prostate miRNAs (54 miRNAs) that were extracted from the literature, which gave 92% classification accuracy. Randomly generated sets of miRNAs of size 50 gave an average accuracy of 63%. To further validate the robustness of the high influence miRNAs, we extracted their expression profiles from two independent prostate miRNA expression profiling studies (NCI60,GSE23022) and showed that predicted high influence miRNAs are accurate prostate cancer biomarkers. Both the high influence miRNA and experimentally verified prostate miRNA lists performed equally well on the NCI60 data (91% classification accuracy), but high-influence miRNAs performed better than known prostate miRNAs on GSE23022 (87% and 77%, respectively). Randomly generated lists of the same size gave an average of 57% accuracy for NCI60 and 52% for GSE23022. Third, predicted functional target modules of the high-impact miRNAs are associated with multiple cancer pathways and prostate cancer related pathways, like TGF-B signaling pathway, and they are involved in several other cancers like glioma, melanoma and bladder (Figure 6). Also they were highly enriched with cell motion and cell migration GO terms (corrected P-value < 0.0005). Pathway enrichment map [50] was used to show the map of enriched biological concepts. This potentially indicates that these genes are important in prostate metastasis. We further extracted 50 prognostic miRNAs from the Taylor data that are associated with aggressive prostate cancer by grouping prostate samples into aggressive prostate cancer (cluster 5 in Taylor study) as one group and the other clusters as the other group. The Top 50 differentially expressed miRNAs were extracted using SAM. 21 out of our 70 high-influence miRNAs were in common with aggressive miRNAs from the Taylor data (p < 0.0001).

Since TargetScan and FPI interaction data are noisy, we repeated the experiments using a highly curated human signaling network and curated miRNA-target interactions from miRecord and miRTarBase. The predicted interaction network (Additional file 1: Figure S8) was found to be modular and partners of miRNA targets are found in highly dense network regions. 31 miRNAs were identified to have high influence on protein signaling network. 27 of them are in the list of 54 prostate miRNAs (p = 0.0001) we collected from literature (Additional file 1: Table S1). Although the topological structure of the target modules are not very similar to modules in Figure 5 due to difference in the protein networks and miRNA-target interactions used to find them, the miRNA targets’ partners are modular and form subnetworks of potential dysregulated proteins. 13 of the 31 miRNAs predicted to have high influence on signaling network were in common with the 70 miRNAs predicted to have high influence on the FPI network (p = 0.0003). These results suggest that the completeness of protein interactions network plays a crucial role to identify high influence miRNAs.

### Functional miRNA-target modules are prognostic biomarkers that help identify patients with aggressive tumors

**Figure 7:**
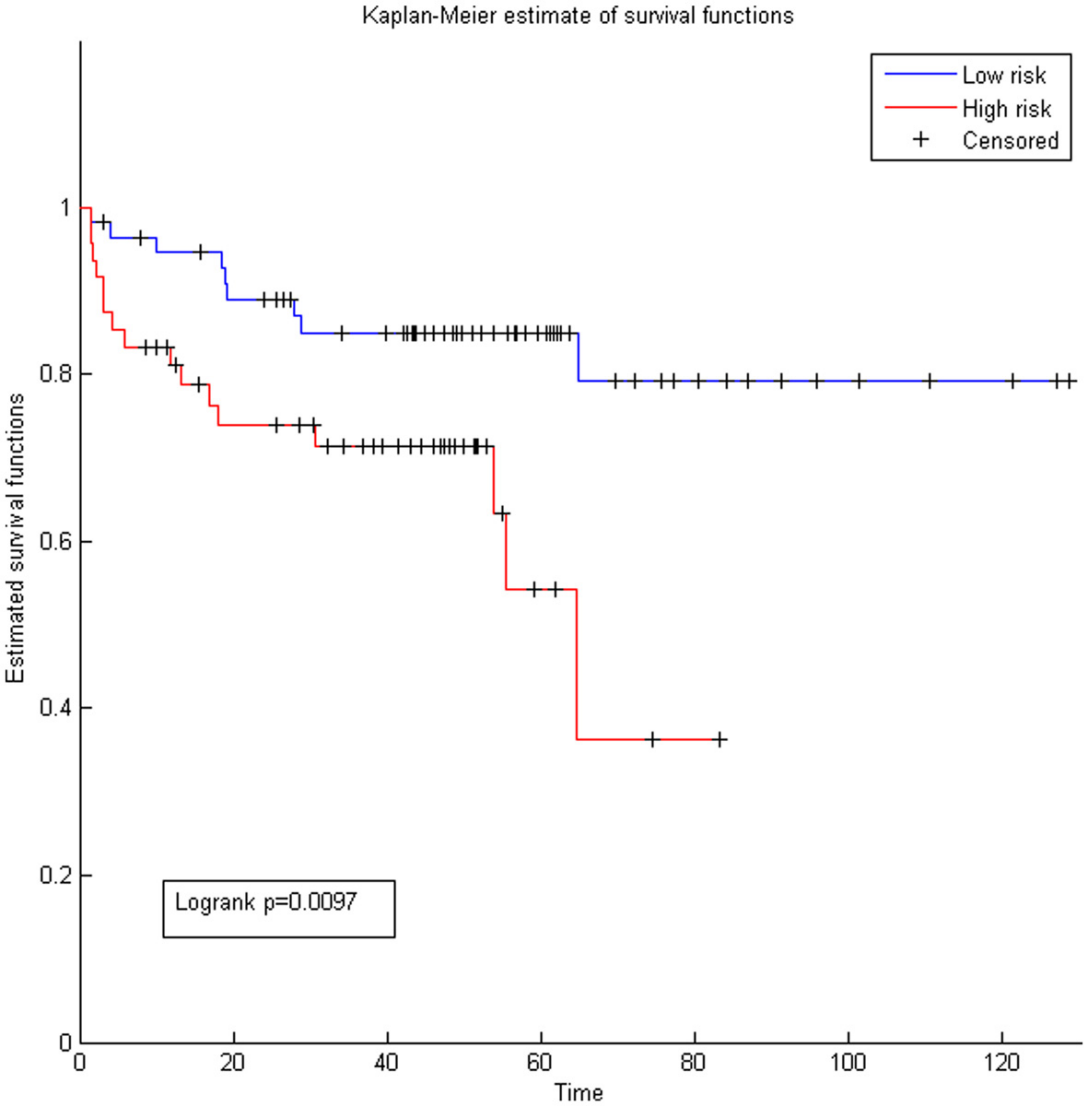
**Kaplan -Meier survival of high-influence miRNAs.** KM are shown for patients classified according to the expression of the functional miRNAs identified in Figure 4. The expression of the functional miRNAs was extracted from the Taylor miRNA data and hierarchical clustering was used to group patients into high and low risk categories. Results revealed that functional miRNAs could be prognostic biomarkers.

We investigated the relationship between the prostate cancer expression profile of miRNAs and outcome using Taylor expression data. We used hierarchical clustering to group patients into two groups (high vs. low risk) based on the miRNA expression profile across all samples. Our findings indicate that the predicted high influence miRNAs are significantly associated with cancer recurrence (Figure 7, logrank p = 0.0097, HR: 2.8). We compared the prognostic effectiveness of the high impact miRNAs with three miRNA lists. The first is miRNAs with experimentally validated targets in prostate cancer (logrank p = 0.015, HR:1.4) (Additional file 1: Figure S3). The second is differentially expressed miRNAs between normal and primary prostate identified from Taylor miRNA expression data (logrank p = 0.019, HR: 1.3) (Additional file 1: Figure S4), and the last list is aggressive prostate specific miRNAs identified from Taylor data (logrank p = 0.00046, HR:3.1) (Additional file 1: Figure S5). Randomly generated lists of miRNAs performed poorly to separate high vs. low risk patients (logrank p = 0.7, HR:0.9). The aggressive prostate specific miRNAs are the most accurate miRNAs to predict cancer recurrence; however, their mode of action is unclear. The identified high influence miRNAs, which have a predicted mode of action (Figure 5) based on our method, were effective at separating high vs. low risk patients (p = 0.0097). We then used target expression from Taylor data to find association between cancer recurrence and target expression, and we used target expression from the Swedish prostate cohort (GSE8402 [51]) to find association between targets and cancer specific death. Results revealed that targets were associated with both recurrence (logrank p = 0.001, HR:2.4) and cancer specific death (0.0034, HR:1.9). Unfortunately miRNA expression is not available for the Swedish cohort, so we could not conduct association between cancer specific death and miRNAs. Altogether, the above results show that the miRNA-target modules, which are the set of proteins targeted by one or more miRNAs, are significant prognostic modules for prostate cancer.

### Patient specific miRNA influence helps predict cancer recurrence

**Figure 8:**
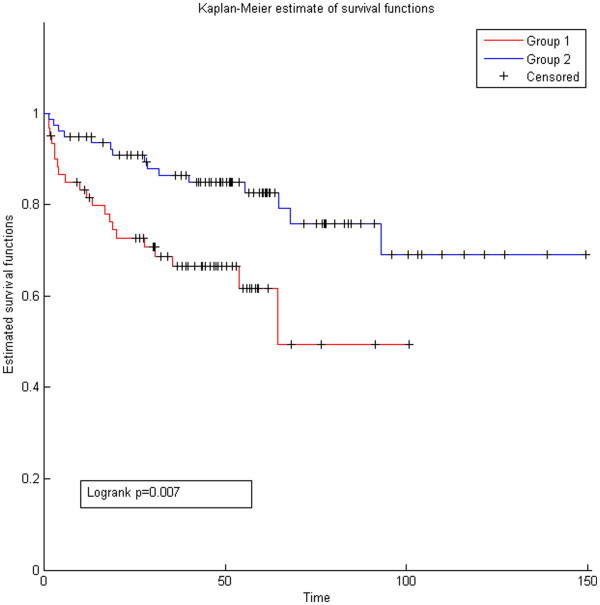
**Kaplan-Meier survival plots for disease-specific cancer recurrence.** KM curves are shown for patients classified according to the influence of the miRNAs on the expression profile of each patient. The influence is predicted using the regression model.

We next investigated if patient specific miRNA influence has prognostic value for cancer recurrence. Using the *miRTI* network and the gene expression profiles of patients as input to our regression model in Eq.(3), we predicted the influence of each miRNA on the expression profile of each patient based on the influence of each miRNA on its targets. As a result, we obtained a patient specific miRNA influence matrix. We used this matrix (miRNA-patient) to identify clinically distinct patient groups. We also used the Seq network instead of *miRTI* as input to the regression model for comparison. We found that using *miRTI* to predict patient specific miRNA influence (Figure 8, logrank p = 0.007, HR:2.2) is substantially more informative than using the binary Seq network (logrank p = 0.507, HR:0.7) (Additional file 1: Figure S6) in predicting cancer recurrence. This further supports our results showing that considering protein interaction context to assess the clinical value of miRNA is informative.

### Identification of miRNAs of high-influence on principal regulators

**Figure 9:**
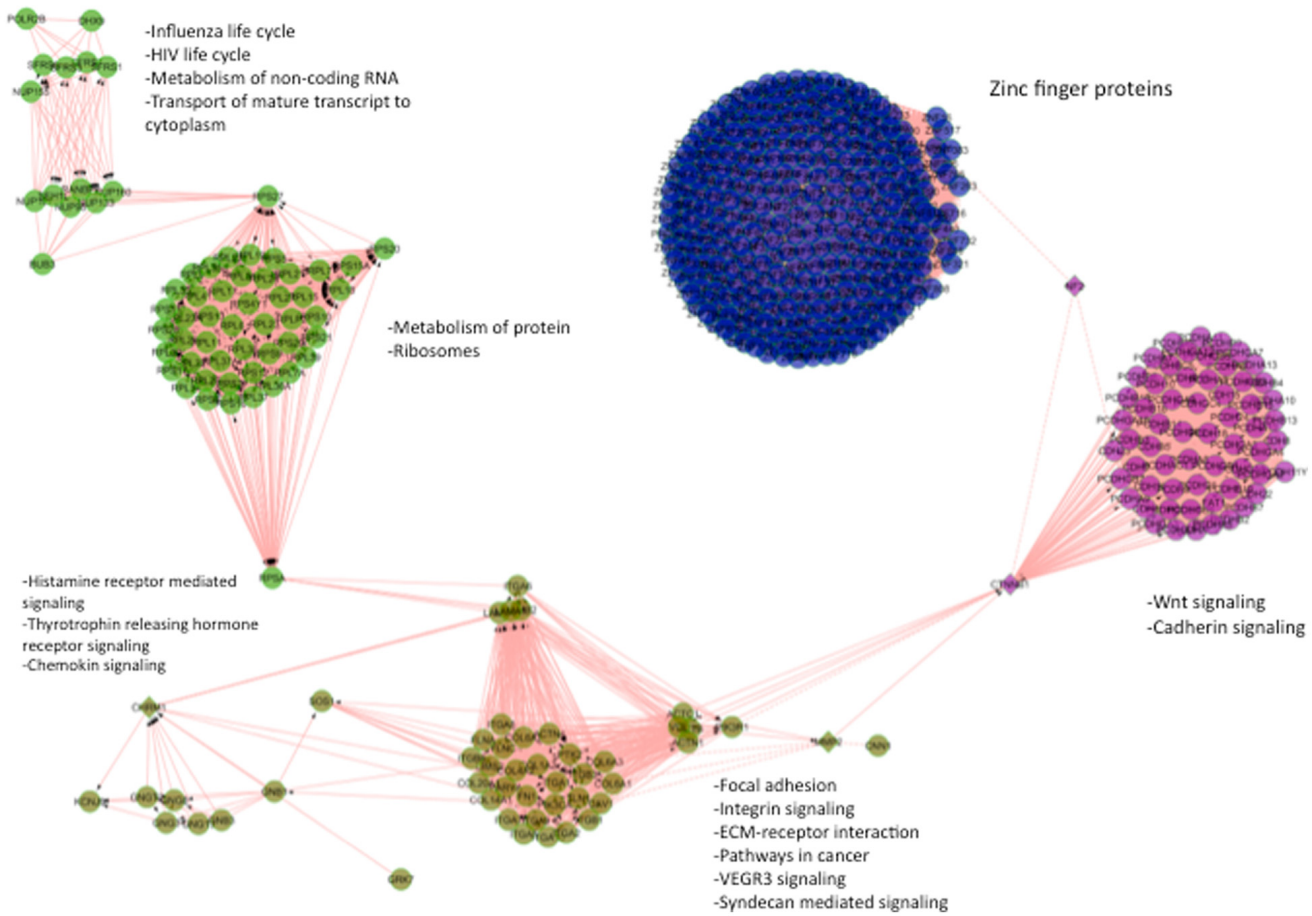
**Interaction network of genes with high ActivityScore.** Network of genes with high ActivityScore indicates that these genes are functionally related and they are highly associated with each other. Cytoscape was used for network visualization.

We next used the *miRTI* network to identify miRNAs that can explain the *ActivityScore* of the target genes. In the previous section, we used the *miRTI* network to predict the gene expression profile of prostate cancer samples. In this section, we propose another application for the *miRTI* network to identify miRNA influence on target gene expession. Integrating biological networks proved effective to help identify biomarkers that can explain most of the gene expression change [52]. *ActivityScore* for each gene is calculated based on the observed effect (*R*) of its neighbors; genes that have high *ActivityScore* are defined as principal regulators. We observed two sets of principal regulators; one with significant *P* and one with non-significant *P* (Additional file 1: Figure S7). The former set represents genes that are significantly differentially expressed (*DE*) and their neighbors are also *DE*. These might be transcription factors that are affected in prostate cancer and they affect their target genes. The latter set represents genes that are non−*DE* but their neighbors are *DE*. These genes might be regulated at the post-translational level instead of at the transcriptional level. For example, a change in the phosphorylation status of a gene might change its activity and thus affect the gene expression of its downstream genes. The functional protein network of the latter set was enriched in zinc-finger proteins (p = 2 × 10^−18^), which are known transcription factors. Figure 9 shows highly connected clusters of proteins of *ActivityScore* greater than the average score of all genes and have p-value greater than 0.05. These proteins are enriched in Wnt and cadherin pathways and focal adhesion (corrected p < 0.005). After calculating the activity score for each gene, we used the *miRTI* network to predict the *ActivityScore* profile for all genes using our regression model in Eq.( 6). Results revealed that miR-221, miR-222, miR-210, miR-542-5p, miR-96, miR-182, and miR-143 are the miRNAs that can positively explain the gene activity profile; this means that increasing expression level of miRNAs will lead to increasing the transcription of activity centers. miR-221 and miR-222 have been characterized as ongogenes [5] and this supports the positive association between the two miRNAs and *ActivityScore*. miR-128 and miR-18b negatively explain the expression profile of the *ActivityScore* of genes which will have negative effect of activity centers. This suggests that these miRNAs might act as oncogenes or tumor suppressors.

Since tumor heterogeneity affects the identification of robust cancer biomarkers, Li et al. [53] found that most cancer gene signatures are not robust and not reproducible. Thus they proposed a re-sampling based framework to identify robust cancer biomarkers. In this work we asked the question whether re- sampling might have an effect on the *ActivityScore* profile. To answer this question, we used Significance Analysis of Microarray (SAM) [54] that is based on re-sampling to identify differentially expressed genes and then generate an *ActivityScore* profile using SAM results. We repeated SAM analysis 100 times; each time we change the permutation number and generated an *ActivityScore* profile. The resulting profiles demonstrated very significant correlation (*R*^2^ = 0.9996) which indicates that re-sampling does not affect the *ActivityScore* and that identified activity centers are robust and reproducible within our data set.

## Discussion

Prostate cancer is one of the most commonly diagnosed malignant tumors in aged men in North America. miRNAs that are a family of regulatory molecules are significantly altered in prostate cancer [5]. However, miRNA’s mode of action and how the influence of prostate miRNAs on target expression is involved in prostate cancer progression is not well known. Over- or under-expression of specific miRNAs in different tumors makes them potential therapeutic targets and diagnostic or prognostic biomarkers; however, miRNAs that are differentially expressed and influence their targets and target partners are important regulators and thus are more promising for diagnostics, prognostics or therapy.

In this work we use functional protein interactions to identify miRNAs with high influence on targets and their partners. We hypothesize that miRNAs that influence a large number of interacting proteins are more important than those that only affect a few proteins. We first showed that proteins that are highly connected have more regulating miRNAs compared to those with low connectivity. Thus, identifying miRNAs that regulate highly connected proteins is important to understand how to control propagation of gene expression changes via miRNAs. We showed that miRNAs that have been experimentally verified to play a role in prostate cancer target functionally related genes. This motivated us to investigate how miRNAs that have high influence on protein partners of the target genes help us to better understand prostate cancer. In this work we bridge a gap between systems biology and clinical biology by investigating the association between miRNAs that have high influence on the system with the outcome of the system.

We built a miRNA-target influence network (*miRTI*) by following miRNA influence of expression in prostate cancer of downstream genes in the FPI network and then proposed three applications of this network. First, we used it to identify miRNA target functional modules and complexes. This revealed miRNAs with high-influence on the target FPI neighborhood, which suggests that these miRNA are important in prostate cancer. The difference between high-influence miRNAs and differentially expressed miRNAs is that high-influence miRNAs are differentially expressed and have differentially expressed targets and target interaction neighbors. Validating both miRNA and targets in the functional modules against independent miRNA expression datasets from prostate indicates that they are robust prostate cancer diagnostic biomarkers. Analyzing functional modules of miRNA targets revealed several results. First, target genes are enriched in prostate cancer and focal adhesion pathways, which may help explain the progression and metastasis process as our data includes metastatic samples. Functional modules are also of prognostic significance as they were associated with cancer recurrence and cancer specific death. Moreover, *miRTI* network (Figure 4) revealed that some proteins like *BTBD*7,*ANK*2,*COL*12*A*1 are highly repressed by several miRNAs. On the other hand, some miRNAs (miRNA-96, miRNA-182, miRNA-1) are highly influential on target partners as they regulate several connected proteins. This suggests that miRNAs have different mode of actions based on their influence on the expression of the target neighborhood. This might help to define new regulatory classes of miRNAs based on their mode of action.

The second application of *miRTI* is to predict patient-specific miRNA influence by using a regression model. In this application we used the *miRTI* network to predict the gene expression profile of the patients (PCs). As a result of the regression model, we predict miRNA-PCs network that shows how much each miRNA explains the gene expression profile of a patient based on the weight with which it affects its targets. We applied the regression model on all patients and generated a matrix that represents the influence of each miRNA on each patient. Based on this miRNA influence matrix we were able to group patients into aggressive and low risk cancer patients. Comparing the *miRTI* with the *Seq* network demonstrated that using miRNA-target influence interactions gives more knowledge about miRNA mode of action than using the binary Seq weights that are based on only sequence predictions. This result supports our initial conclusion that considering the downstream effect of miRNA on protein partners of target is useful and has prognostic value. We realized that both grouping patients based on miRNA gene expression and based on patient-specific miRNA influence from miRNA-PCs network result in putting high risk patients in one group and low risk patients in the other group. This indicates that the influence of each miRNA on each patient is represented in the mRNA expression of the patient. The availability of differential miRNA and mRNA expression profiles from the same cancer samples enable functional analysis of miRNAs in cancer, but there are few cancer cohorts that have expression levels of miRNA and mRNA from the same sample. Thus this result is very promising to predict the expression of miRNAs in patients and predict their outcome without performing miRNA expression profiling.

The third application of the *miRTI* network is to predict miRNAs with high-influence on genes with high activity center scores (highly active network neighborhoods). The *ActivityScore* profile of prostate cancer summarizes the activity of module proteins rather than the activity of single genes as in the second application. Here the *miRTI* is used to predict the *ActivityScore* using the regression model. The results emphasized the role of some miRNAs already validated in prostate cancer (miR-221, miR-222, mir-96 and mir-143), and identified novel miRNAs like miR-210, miR-542, miR-128 and miR-219 that do not have a known mode of action in prostate cancer. This means that these miRNAs could be as important as the already validated miRNAs, and could explain the summarized activity of the gene modules. miRNAs identified using the *miRTI* and *Corrmir* networks overlap; both networks identified miR-182 and miR-96 as important miRNAs. The advantage of using *miRTI* over *Corrmir*, *Seq* and *W * to identify miRNA influence on target partners or on patient gene expression is that it produces two types of modules, unlike *W * that favors the first type of modules and *Corrmir* that favors the second type of module. Modules identified by our approach includes miRNAs like miR-96 and miR-182 targeting highly interacting proteins, and miRNAs like miR-1, and miR-205 that target non-interacting complexes.

miRNAs have been associated with clinical variables, prostate cancer recurrence and prostate cancer-specific death [55]. However, the association between miRNAs that target protein modules vs. clinical and survival data has not been well studied. Recent evidence showed that low miR-1 in human prostate tumors is associated with early disease recurrence [56], and elevated levels of miR-96 is associated with high Gleason score and higher risk of biochemical relapse [55]. In this work we showed that miRNAs identified using the *miRTI* method are associated with cancer recurrence (Figure 7). Also, we showed that patient-specific miRNA influences predicted using *miRTI* are better prognostic biomarkers compared with binary, non-weighted miRNA-target interactions. This indicates that there is a link between the influence of miRNA on target partners and its influence on outcome, but more analysis on larger cohorts and biological experiments are required to prove this result.

Comparing the three applications of *miRTI* revealed consistent results. They all indicate the significant role of specific miRNAs (miR-221, miR-222, miR-210, miR-542-5p, miR-96, miR-182, and miR-143) in prostate cancer. For instance, miR-96 and miR-182 are members of the same gene cluster and thus this supportes the effectiveness of integrating protein networks to identify miRNAs with similar mode of action. *ActivityScore* functional analysis indicates that zinc-finger proteins, zinc homeostasis, focal adhesion, and Wnt signaling are enriched in genes with high *ActivityScore* (p-value < 1 × 10^−10^). Evidence showed that zinc homeostasis is regulated by the miR-96-183-182 cluster. This is in agreement with our results that demonstrate that miR-96 and miR-182 explain most of the genes *ActivityScore* that is significantly enriched in zinc homeostasis. Other predicted miRNAs (miR-143, miR-542) may play a role in zinc homeostasis, focal adhesion, and cytoskeleton organization.

The large scale protein interactions and miRNA target prediction data we used were useful to help elucidate the mechanistic role of miRNAs in disease progression. Although the interaction datasets are far from complete and suffer from noise, our results were consistent across choice of PPI network. Using additional protein interaction networks, different miRNA target prediction algorithms, and different expression data sets will likely reveal more miRNAs with high-influence on cancer progression. Another future direction for this work is designing a systematic method to combine the three variables that determine the influence of miRNAs on the target partners.

Finally, this study on bridging the gap between clinical bioinformatics and network-based biomarkers provides clear evidence that protein interaction information is useful to identify diagnostic and prognostic cancer biomarkers, and to ameliorate the understanding of the functional mechanisms of miRNAs.

## Conclusion

We have developed a novel method to identify active miRNA-target modules relevant to prostate cancer progression and outcome. miRNAs with high influence on protein networks are valuable biomarkers that can be used in clinical investigations for prostate cancer treatment. Combining the effects of miRNAs on targets and target partners provides better understanding of miRNAs function.

## Competing interests

The authors declare that they have no competing interests.

## Authors’ contributions

MA, GB and AG designed the study. MA wrote the initial manuscript. GB edited the manuscript. QM and RA proof-read the manuscript. All authors contributed to the writing and approved the final manuscript.

## Supplementary Material

Additional file 1 **Experimentally validated Prostate miRNAs and supplementary figures.** This file lists miRNAs that have been studied in prostate cancer and showed to play a role in prostate cancer progression. It also shows some targets of these miRNAs that have been validated in prostate cancer context. Click here for file
