# Supplementary material for: Detecting microRNAs of high influence on protein functional interaction networks: a prostate cancer case study
Source: BMC Syst Biol. 2012 Aug 28;6:112. doi: 10.1186/1752-0509-6-112 (PMC3490713; doi:10.1186/1752-0509-6-112)
Supplement: Additional file 1 — Experimentally validated Prostate miRNAs and supplementary figures. This file lists miRNAs that have been studied in prostate cancer and showed to play a role in prostate cancer progression. It also shows some targets of these miRNAs that have been validated in prostate cancer context. [file 1752-0509-6-112-S1.pdf]

Detecting microRNAs of high influence on protein functional interaction networks: a prostate cancer case study

Mohammed Alshalalfa, Gary D.Bader, Anna Goldenberg, Quaid Morris, Reda Alhajj

Table S1: This table lists the miRNAs that we predict play a role in prostate cancer progression based on our analyzed expression data sets; either prostate cell lines or prostate tissue. It also lists the experimentally verified targets for some of them that we extracted from literature.

miRNA	Known targets in prostate context (cell line or tissue)	Reference	Function	Predicted High-influence on protein networks	Experimentally verified target pathways
miRNA-221	$P27^{Kip1}$ (CDKN1B)	1,8	Oncogene	*	Development and metastasis of PCa, AR–mediated signaling
miRNA-222	$P27^{Kip1}$ (CDKN1B)	1,8	Oncogene		Development and metastasis of PCa, AR–mediated signaling
miRNA-21	MARCKS<br>PDCD4<br>TPM1<br>BMPRII	1,8<br>1,8<br>1,8<br>3,8	Oncogene	*	Development and metastasis of PCa , AR-mediate signaling
miRNA-125b	BAK1<br>EIF4EBP1	1,8<br>1	Oncogene	*	cell proliferation
miRNA-101	EZH2	1,3,8	Tumor suppressor	*	Cell growth and invasiveness
miRNA-126*	Prostein (SLC45A3)<br>EGFL7	1,8<br><br>2	Tumor suppressor		VEGF signaling and vessel development
miRNA-146a	ROCK1	1,3	Tumor suppressor		HA/Rock1 pathway

miRNA-330	E2F1	3	Tumor suppressor	*	AKT signaling
miRNA-200a	ZEB1<br>ZEB2	3<br>3	Tumor suppressor		epithelial to mesenchymal transition
miRNA-200b	ZEB1<br>ZEB2	3<br>3	Tumor suppressor		epithelial to mesenchymal transition
miRNA-429		3	Tumor suppressor		epithelial to mesenchymal transition
miRNA-200c	ZEB1<br>ZEB2	3,8			
miRNA-141		3			
miRNA-34a	P53<br>CDK6<br>SIRT1	3,8<br>3,8<br>3,8	Tumor suppressor	*	AR, p53 pathway
miRNA-17-5p		3	Tumor suppressor		
miRNA-17-3p	VIM	3	Tumor suppressor		
miRNA-193b		8		*	
miRNA-92		3			
miRNA-106a		3,8		*	
miRNA-155		3,8		*	
miRNA-338		3		*	
miRNA-126		3			
miRNA-181b		3,8			
miRNA-181c		3			
miRNA-224		3,8			
miRNA-23b	c-MYC<br>GLS	3			
miRNA-100	SMARCA5<br>SMARCD1<br>mTOR	3,6			
miRNA-145	ICP4<br>MYO6	3,4,8		*	
miRNA-15a	BCL2<br>CCND1<br>WNT3A	3	Tumor suppressor	*	VEGF signaling
miRNA-16	BCL2<br>CCND1<br>WNT3A	3,8	Tumor suppressor	*	VEGF signaling
miRNA-23a	c-MYC	3	Tumor		

	GLS		suppressor		
miRNA-449a	HDAC1	3	Tumor suppressor		
miRNA-331-3p	CDCA5<br>KIF23<br>ERBB2	3<br>3<br>3,8	Tumor suppressor		
miRNA-373	CD44	3	Tumor suppressor		
miRNA-520c	CD44	3	Tumor suppressor		
miRNA-205	ZEB2<br>ERBB3<br>PRKCE	3,8<br>3,8<br>3,8	Tumor suppressor	*	
miRNA-143	ERK5<br>MYO6<br>ICP4	3,8<br>8<br>8	Tumor suppressor	*	
miRNA-20a	E2F1<br>E2F2<br>E2F3	3<br>3<br>3	Oncogene	*	
miRNA-106b	E2F1<br>CDKN1A	3<br>3	Oncogene	*	
miRNA-32	Bim1<br>BCL2	3	Oncogene		
miRNA-218		3		*	
miRNA-let7c		3,8		*	
miRNA-1	HDAC4<br>XPO6<br>Notch3	4	Tumor suppressor	*	Inhibiting cell proliferation
miRNA-206	HDAC4	4			
miRNA-133a		4			
miRNA-182	SLC39A1	5		*	
miRNA-183		5			
miRNA-96		5		*	
miRNA-99a	SMARCA5<br>SMARCD1<br>mTOR(FRAP1)<br>KLK3	6<br>6<br>6			
miRNA-99b	SMARCA5<br>SMARCD1<br>mTOR (FRAP1)<br>KLK3	6			
miRNA-204		7			
miRNa-210		7			
miRNA-451		7		*	
mi-RNA425-5p		7			

*: indicates that the miRNA is predicted to have high influence on target partners based on miRNA-target influence network

Reference:

[1] Pang Y , Young CY and Yuan H, MicroRNAs and prostate cancer, Acta Biochim Biophys Sin, 2010, 42:363-369

[2] Nikolic I, Plate K, Schmidt M, EGFL7 meets miRNA-126: an angiogenesis alliance, Vascular Cell, 2010, 2:9

[3] Saini S, Majid S, and Dahiya R, Diet, microRNAs and prostate cancer, Pharm Res, 2010, 27:1014-1026

[4] Hudson PS, et al, MicroRNA-1 is a candidate tumor suppressor and prognostic marker in human prostate cancer, Nucleic Acids Research, 2011, 1-15

[5] Mihelich BL, et al, miR-183-96-182 cluster is overexpressed in prostate tissue and regulates zinc homeostasis in prostate cells, The J.of Biol Chem, 2011, 296:44503-44511

[6] Sun D, et al, miR-99 family of microRNAs suppresses the expression of prostate-specific antigen and prostate cancer cell proliferation, Cancer Research, 2011, 71:1313-1324

[7] Martens-Uzunova ES, et al, Diagnostic and prognostic signature from the small non-coding RNA transcriptome in prostate cancer, Oncogene, 2012, 31:978-991

[8] Xu J et al, Prioritizing candidate disease miRNAs by topological features in the miRNA target-dysregulated network: case study of prostate cancer, Molecular Cancer Therapeutics, 2011,10,1857-1866

Supplementary Figures

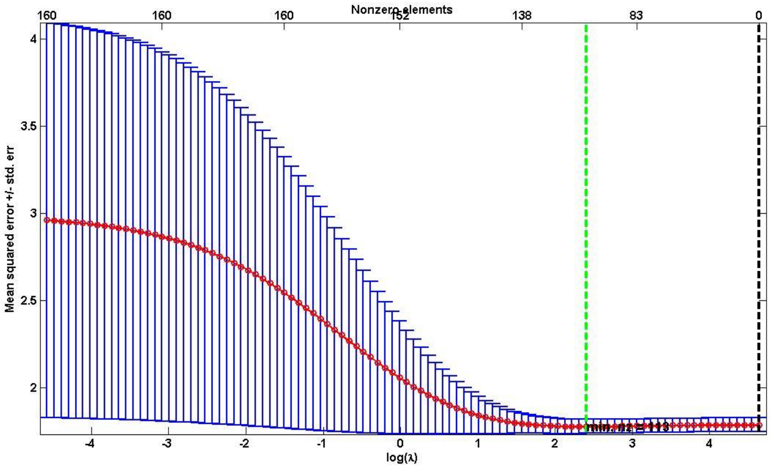

A

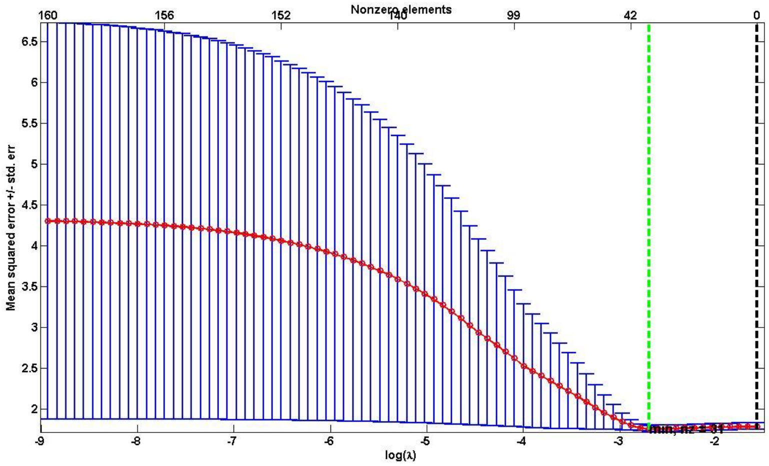

B

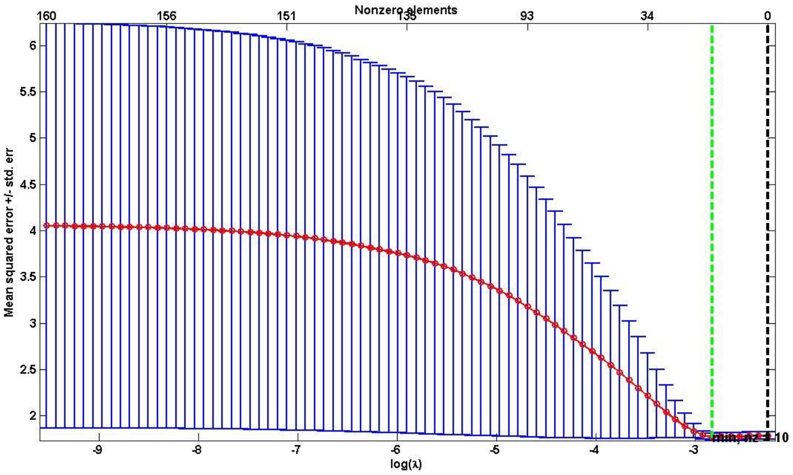

C

Figures S1**: Lambda optimization with respect to different alpha values**. (A) Alpha is set to 0 in this scenario. Number of non zero elements is 113 out of 161 predictors and Lambda value is 11. (B) Alpha is set to 0.5. Number of nonzero elements dropped to 31 which increase the sparsity of the solution and Lambda values is 0.11 in this scenario. (C) Alpha is set to 1. This reduced the non zero elements to 10 with very sparsed solution that may lead to loss of lots of potential predictors and lambda 0.04.

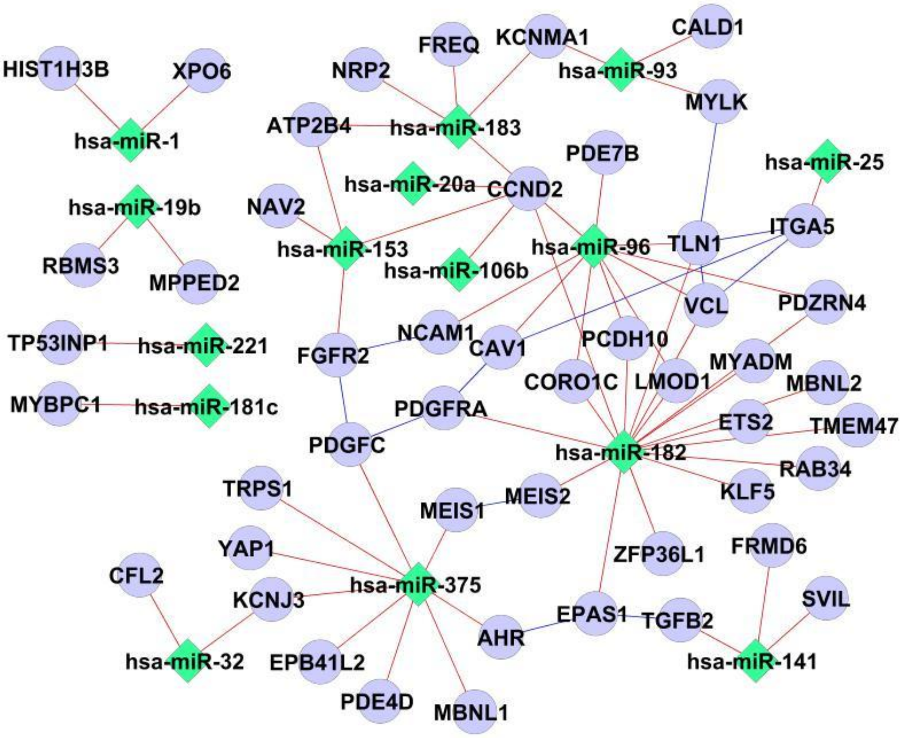

Figures S2: **Correlated miRNA-target network extracted based on correlation between a miRNA expression profile and its targets without including protein network information**

The network shows that not considering the downstream regulation of miRNAs on the FPI network leads to a substantial loss of influence relationships, including a large one targeting a dense network region corresponding to the focal adhesion complex. Cytoscape was used for network visualization.

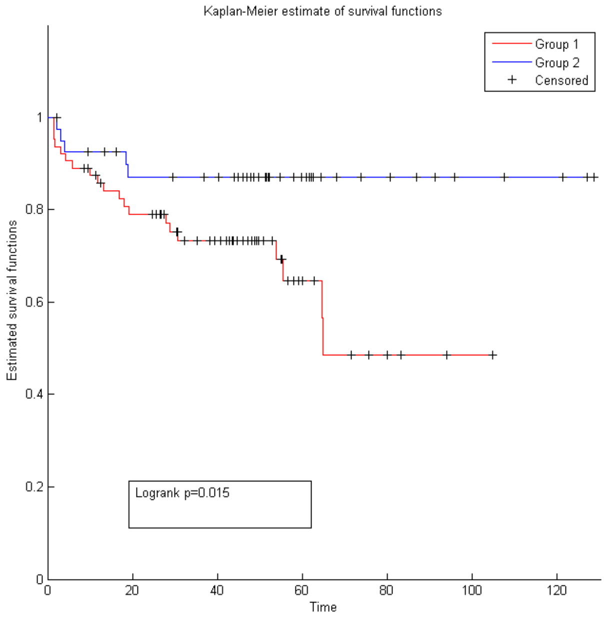

Figure S3: **Kaplan Meier curves for prostate miRNA**

We extracted the expression levels of experimentally verified prostate miRNAs and then grouped samples into two groups.

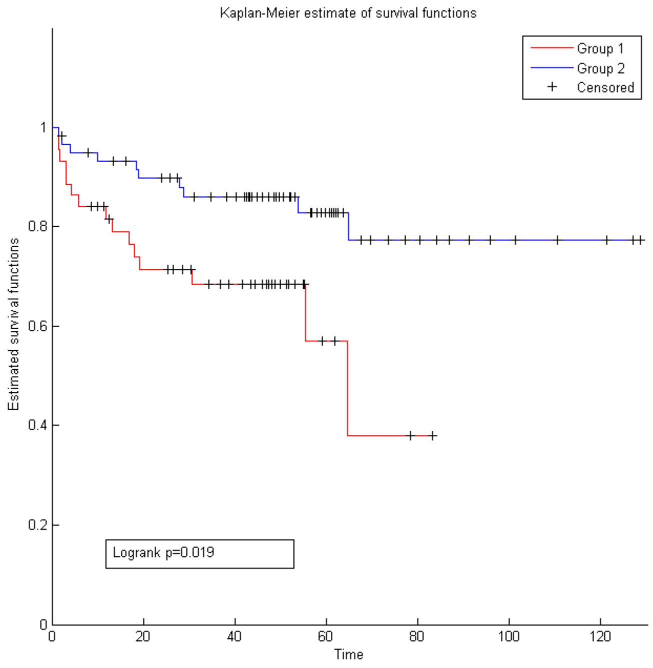

Figure S4: **Kaplan-Meier curve of 94 differentially expressed miRNA in prostate cancer**

We extracted the expression levels of prostate miRNAs that showed differential expression pattern in cancer v normal samples in Taylor data, and then grouped samples into two groups. Results showed that diagnostic biomarkers can act as prognostic biomarkers too.

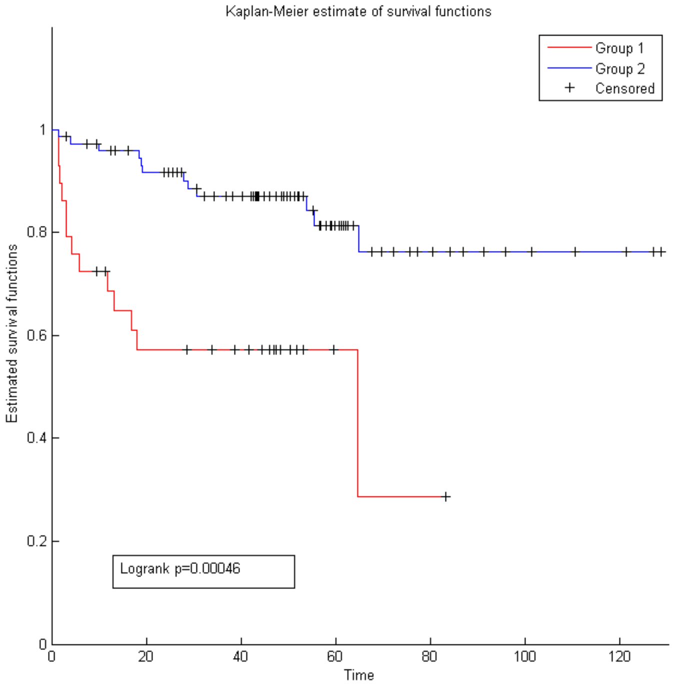

Figure S5: **Kaplan-Meier curve of 50 gene signature of aggressive prostate cancer**

We first extracted 50 gene as a signature of aggressive prostate cancer from Taylor data (t-test $p<0.001$). We extracted their expression from Taylor data , and then grouped samples into two groups. Results showed that aggressive cancer miRNAs are accurate prognostic biomarkers.

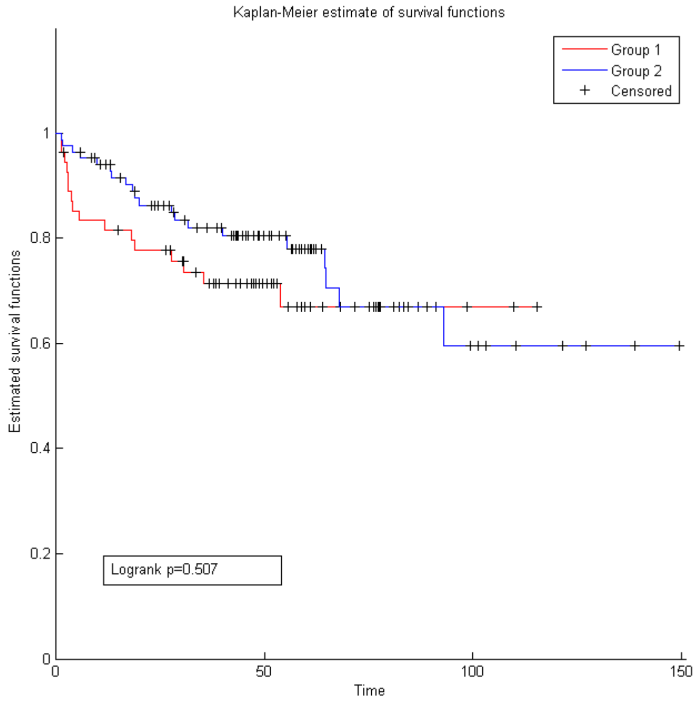

Figure S6: **Kaplan -Meier survival plots for disease-specific cancer recurrence**

KM curves are shown for patients classified according to the influence of the miRNAs on the expression profile of each patient using Seq data (protein context effect is eliminated). The influence is predicted using the regression model. Results showed that binary association between miRNA and targets is not clinically significant.

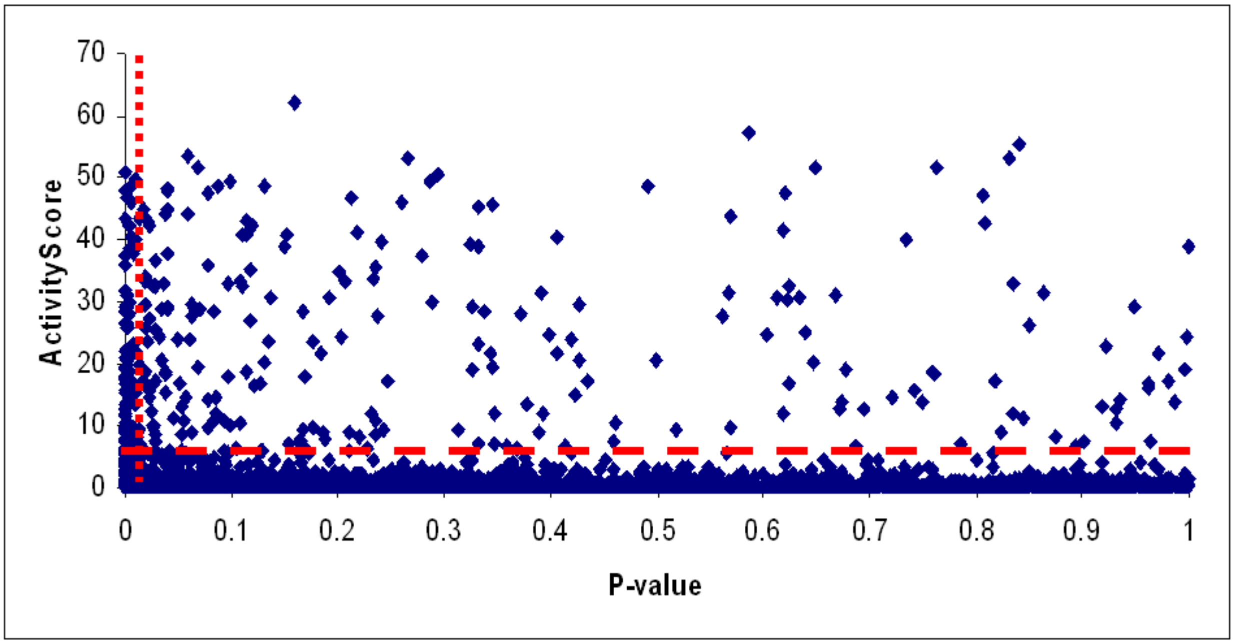

Figure S7: **ActivityScore vs. Student's t-test p-value**

Most genes with high ActivityScore were not significantly differentially expressed. Genes with high ActivityScore and insignificant p-value are further biologically characterized as in Figure 8. These genes are enriched with zinc finger proteins and wnt signaling pathways.

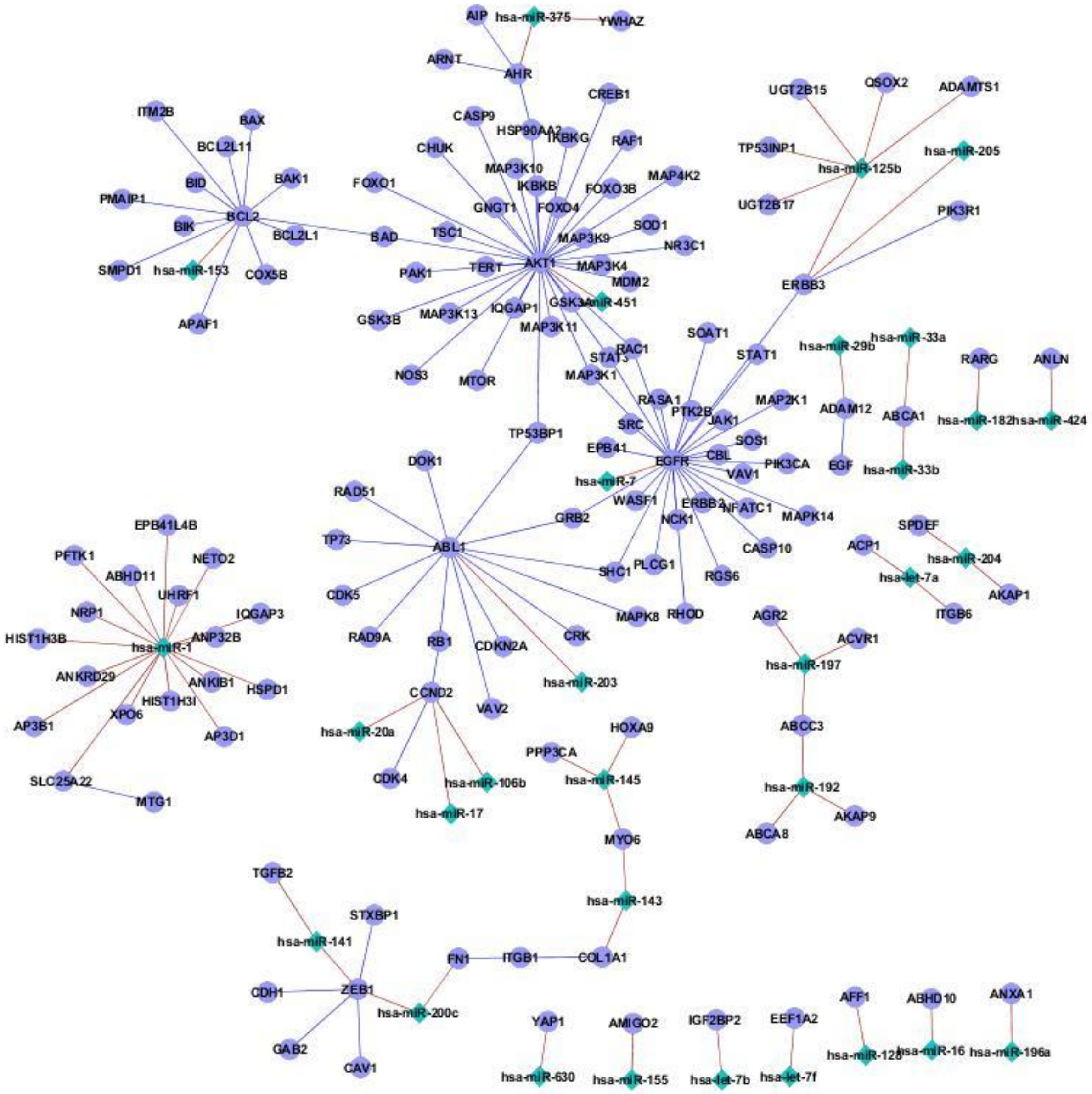

Figure S8: miRNA-target network extracted by using Human signaling network and curated miRNA-target interactions from miRecord and miRTarBase
